# Best Practices and Policies for Addressing Social Isolation Among Older Adults

**DOI:** 10.1093/geroni/igaa057.1020

**Published:** 2020-12-16

**Authors:** Raza Mirza, Samir Sinha, Andrea Austen, Anna Liu, Jaemar Ivey, Michelle Kuah, Lynn McDonald, Jessica Hsieh

**Affiliations:** 1 National Initiative for the Care of the Elderly, Toronto, Ontario, Canada; 2 Sinai Health, Toronto, Canada; 3 City of Toronto, Toronto, Ontario, Canada; 4 Ontario Ministry of the Environment, Toronto, Canada; 5 University of Toronto, Toronto, Canada; 6 National Initiative for the Care of the Elderly, Toronto, Canada; 7 University of Toronto, Toronto, Ontario, Canada

## Abstract

Isolation has been flagged as a major health and social problem for seniors. Yet, many seniors themselves, their friends/family, and carers for seniors may not recognize risk factors for isolation or know what to do if a senior is isolated. Results from the 2016 General Social Survey noted that 27% of seniors reported they were not socially connected with others, with 20% reporting that they lacked support to carry out chores, and 17% reported feeling isolated. However, there has yet to be a comprehensive review of the evidence to suggest what has emerged as best practices and key policy enablers. To address this, seniors and other key stakeholders (n=200) in the community were interviewed on their perspectives and experiences of social isolation. Additionally, three focus groups (n=24) were conducted, along with a consensus meeting, to identify top priorities, best practices and develop implementation strategies. The priority areas identified were: 1) opportunities for seniors to network and be part of the social fabric; 2) initiatives promoting inclusive community development; 3) programs that promote education related to social isolation; 4) develop services that place an emphasis on partnerships/collaborations; 5) services that are sustainable over the longer term. By mapping the best, emerging practices and policies for social isolation, the ability to synthesize the evidence on social isolation and co-create knowledge translation tools with seniors and other stakeholders will be possible. This will help identify solutions and policies that can be used by governments, health systems, and individuals to comprehensively target social isolation.

